# *PSIP1/LEDGF*: a new gene likely involved in sensorineural progressive hearing loss

**DOI:** 10.1038/srep18568

**Published:** 2015-12-22

**Authors:** Giorgia Girotto, Déborah I. Scheffer, Anna Morgan, Diego Vozzi, Elisa Rubinato, Mariateresa Di Stazio, Enrico Muzzi, Stefano Pensiero, Anne B. Giersch, David P. Corey, Paolo Gasparini

**Affiliations:** 1 University of Trieste-Department of Medical, Surgical and Health Sciences, Trieste, Italy; 2 Harvard Medical School-Howard Hughes Medical Institute, Department of Neurobiology, Boston MA, United States; 3 Medical genetics, IRCCS Burlo Garofolo, Trieste, Italy; 4 Audiology and Pediatric Otorhinolaryngology, IRCCS Burlo Garofolo, Trieste, Italy; 5 Department of Ophthalmology, IRCCS Burlo Garofolo, Trieste, Italy; 6 Harvard Medical School and Brigham and Women’s Hospital, Department of Pathology, Boston MA, United States

## Abstract

Hereditary Hearing Loss (HHL) is an extremely heterogeneous disorder. Approximately 30 out of 80 known HHL genes are associated with autosomal dominant forms. Here, we identified *PSIP1/LEDGF* (isoform p75) as a novel strong candidate gene involved in dominant HHL. Using exome sequencing we found a frameshift deletion (c.1554_1555del leading to p.E518Dfs*2) in an Italian pedigree affected by sensorineural mild-to-moderate HHL but also showing a variable eye phenotype (i.e. uveitis, optic neuropathy). This deletion led to a premature stop codon (p.T519X) with truncation of the last 12 amino acids. *PSIP1* was recently described as a transcriptional co-activator regulated by miR-135b in vestibular hair cells of the mouse inner ear as well as a possible protector against photoreceptor degeneration. Here, we demonstrate that it is ubiquitously expressed in the mouse inner ear. The *PSIP1* mutation is associated with a peculiar audiometric slope toward the high frequencies. These findings indicate that *PSIP1* likely plays an important role in HHL.

Hereditary Hearing Loss (HHL) is a common genetic disorder accounting for at least 60% of pre-lingual deafness in children. Most cases (70%) are non-syndromic, thus they are not associated with other signs or symptoms. The most common pattern of inheritance for non-syndromic HHL (NSHHL) is autosomal recessive (DFNB), which accounts for 75–85% of nonsyndromic cases. The other 15–25% have an autosomal dominant (DNFA) inheritance, while the remaining cases (1–2%) have an X-linked (DFN) or mitochondrial inheritance pattern^1^. In 30% of all cases the pattern is syndromic, in which hearing loss is not the only clinical feature. In some syndromic cases, the phenotype combines the presence of hearing loss with vision defects (i.e. Usher, Alport, Wardenburg, Stickler, etc.)^2^ but very few of them are transmitted as dominant traits. HHL can involve loss or dysfunction of cochlear cells and is caused by defects in several different molecular pathways. To date, several hundred mutations in approximately 80 disease-causing genes are known to be associated with NSHHL and many others underlie syndromic forms^1^.

Despite the identification of an increasing number of genes causing hearing loss, a large proportion of cases of DFNA lack a genetic association to one or more of the 30 known DFNA genes^3^. To increase our understanding of the genetics of DFNA, we focused on hereditary cases in which the families did not carry a mutation in any of the previously reported HHL genes. Three Italian families characterized by sensorineural bilateral dominant HHL were enrolled in the study. All families included in the study were negative for mutations in the *GJB2* and *GJB6* genes or for the 1555A > G mitochondrial mutation which are the most commonly mutated genes among Mediterraneans^4^,^5^,^6^. In order to reveal the underlying genetic defect in these families, we performed targeted re-sequencing of known HHL genes following the strategy previously described on affected family members, but failed to identify any pathological mutations^7^. The first large kindred whose affected members showed sensorineural mild-to-moderate hearing loss was further investigated by whole-exome sequencing. Using this approach, we identified a heterozygous frameshift deletion in a transcriptional co-activator, *PSIP1* (also called *LEDGF*), already known to be regulated by miR-135b in vestibular cells^8^ and recently described as a regulator of gene expression in the epithelial cells of the lens^9^. In mouse, we found that *Psip1* expression is constant over the course of cochlear development into adult life and that the protein remains abundant in the mature inner ear with a characteristic location in the nucleus of sensory epithelial cells. Moreover, we found that the frameshift mutation identified in human does not alter the mRNA stability. Our findings identify a new interesting candidate gene for human HHL and possibly visual degeneration as well.

## Results

**Figure 1:**
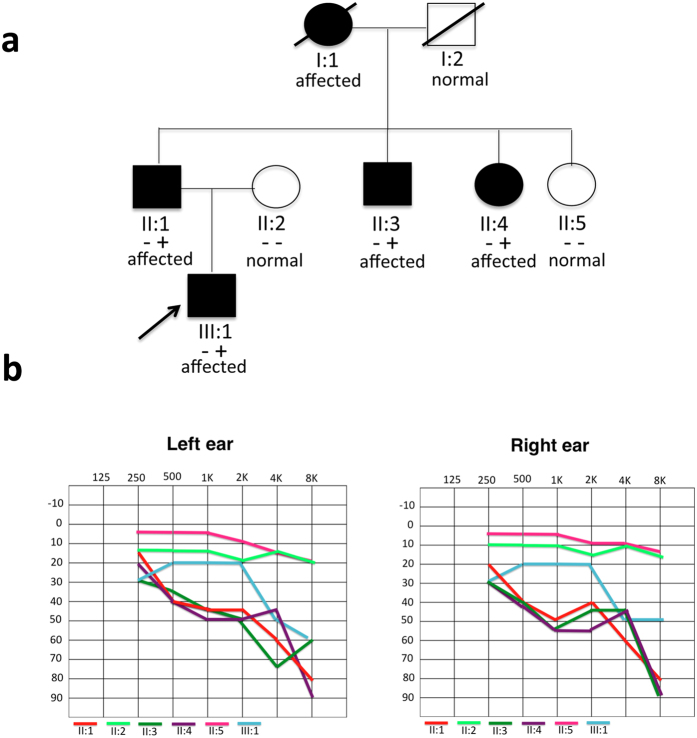
Pedigree and clinical features of the family. (**a**) Pedigree of the Italian family carrying the mutation in the *PSIP1* gene. Filled symbols represent affected individuals. −: homozygous wild type status, +-heterozygous mutated status. (**b**) Audiometric features of the affected and healthy individuals (II:1, II:2, II:3, II:4, II:5, III:1) displayed as pure tone audiograms (air conduction = bone conduction) and showing left and right hearing thresholds. These audiograms represented the latest audiological examination performed in April 2014, when the subjects were respectively: II:1 56 years old (y.o.), II:2 50 y.o., II:3 62 y.o., II:4 67 y.o., II:5 51, III:1 19 y.o. The downsloping threshold indicates that high frequencies are more severely affected than low frequencies .All patients’ Pure Tone Average (PTA, 500–1000–2000 Hz) shows a mild-to-moderate hearing loss.

In this study, an Italian DFNA family showing bilateral progressive mild-to-moderate sensorineural hearing loss with an age of onset ranging from 15 to 40 years old (Fig. 1a,b), without any vestibular dysfunction (assessed by clinical history and thorough bedside examination), was analyzed by whole exome sequencing analysis and found to be mutated in *PSIP1*. Audiometric profiles of affected individuals show a symmetrical mean pure tone average (PTA, 500–1000–2000 Hz) of 40 dB HL, gently-to-steeply sloping towards the high frequencies, characteristic of very few DFNA genes as predicted by the Audiogene software^10^.

To localize the site of lesion along auditory pathways, we performed an Auditory Brain Response (ABR) exam in the proband (III:1). Click-ABR audiometry is an effective tool in the evaluation of downsloping hearing loss, to discriminate between cochlear and retrocochlear involvement. The ABR at 80 dB nHL (stimulus rate: 21.0/s, polarity: rarefaction, sweeps: 1500 per track) showed normal amplitude, morphology and replicability in both ears, without significant changes at high stimulation rates (61.0/s). Absolute, interpeak and interaural latencies of wave I, III and V were within reference values. ABR features are thus consistent with cochlear origin of the hearing loss in the proband (III:1). Because this gene is reported as expressed in retinal photoreceptors^11^, we performed the Visual Evoked Potentials (VEPs) exam in three affected members in order to investigate the functional integrity of the visual pathways from retina via the optic nerves to the visual cortex of the brain. The proband (III:1) showed an idiopathic anterior chronic uveitis, complicated by a bilateral posterior cortical cataract, from the age of 13 years, which had been treated with topical steroids. Electroretinography (ERG) indicated normal rod and cone functionality, and the 6 cycles/deg pattern visual evoked potentials (p-VEPs) test was normal and symmetric for amplitude (14 μV) and P100 latency (124 ms), demonstrating the integrity of the visual pathway up to the visual cortex. Visual acuity was 20/20 in the right eye (RE) and 20/22 in the left eye (LE) with a slight myopic optical correction (−0,50 diopters in both eyes). The proband’s father (II:1) showed a low visual acuity (20/40 in both eyes), with a myopic refraction (−1.75 D in the RE and −2.50 D in the LE), due to a bilateral optic retrobulbar neuritis presenting at 40 years of age. Rod and cone ERG were normal, while p-VEPs were altered, with an amplitude of only 4 μV and a large latency of 170 ms in both monocular stimulations. At fundus examination, a bilateral pale optic disc and a dystrophy of the retinal pigmented epithelium in the RE were observed. The patient is afflicted by hypertension, hyperglycemia and dyslipidemia; he was a smoker until 15 years ago. Finally, patient II:3 showed a myopic anisometropic refraction with a slight reduction of visual acuity. In the RE it was 20/30 with a −4.25 D correction, in the LE 20/25 with −0.75 D. Rod and cone ERG were normal, while p-VEPs showed a reduction of the amplitude (3 μV) and a moderate symmetrical increase of latency (132 ms). No other alteration was evidenced at the ophthalmic examination. These findings are consistent with a bilateral pathology of the optic nerve, probably the outcome of a retrobulbar neuritis, but no indication was supplied by the patient about the clinical history of his reduced visual acuity.

### Mutational screening of known HL-related genes

To reveal the underlying genetic defect in a series of three Italian HHL families, we initially screened 96 genes already known to be related to HHL using our established targeted sequencing approach^7^. Affected members of these families were negative for the presence of mutations in *GJB2* and *GJB6* genes and for the 1555A > G mitochondrial mutation. All families were negative for the presence of mutations in the 96 genes in our hearing loss panel.

### Exome-sequencing detects a frameshift deletion in *PSIP1*

**Figure 2:**
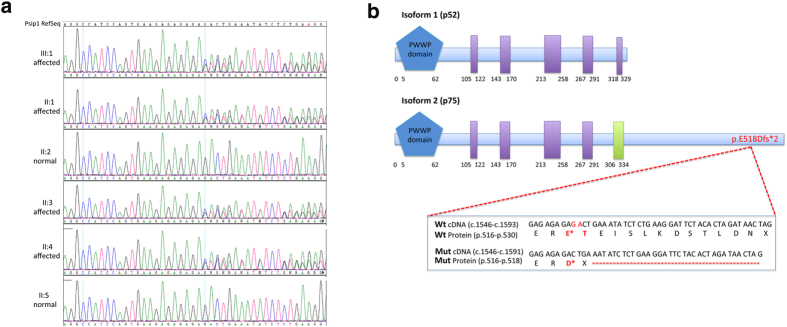
DNA sequence and protein structure of the *PSIP1* gene. (**a**) DNA chromatograms of all the family members. Affected patients (III:1, II:1, II:3, II:4) show the c.1554_1555del mutation at the heterozygous state, while healthy members (II:2, II:5) are wild type. (**b**) Schematic representation of the structure of the two protein isoforms of *PSP1*, p75 and p52. p75 is encoded by exons 1–15 and contains 530 amino acids, while the alternatively spliced p52 is encoded by exons 1–9 and contains 333 amino acids. The two isoforms share a region of 325 amino acids. In blue the PWWP domain, a region with conserved P-W-W-P residues; in purple low complexity regions; and in green a coiled-coil region. The mutation (c.1554_1555del, p.E518Dfs*2) affects only the second isoform and alters the reading frame downstream of codon 518 causing an ammino acid substitution in position 518 (p.E518D) and a premature stop codon (PTC) (p.T519X) with truncation of the last 12 amino acids.

Following our standard gene discovery strategy, we analyzed these three DFNA families by whole-exome sequencing using Ion Proton technology (Life Technologies, CA, USA), starting from the largest kindred available (Fig. 1a). DNAs from affected family members (II:1, II:3, III:1) and an unaffected family member (II:5) were subjected to whole exome sequencing. The overall mean-depth base coverage for sequencing was 108X, while on average 89% of the targeted region was covered at least 20-fold (Table 1). A total of 71,037 genetic variants were called among the four sequenced subjects. In accordance with the variants-filtering procedure described in Methods, we identified 21 candidate variants and, specifically, a likely pathogenic two base-pair frameshift deletion (NM_001128217; c.1554_1555del) in the *PSIP1* gene in the three affected family members. Sanger sequencing on the whole family (six members: four affected and two normal hearing) confirmed this allele as the only one segregating with the hearing loss phenotype within the family (Fig. 2a). It leads to a premature stop codon in the PSIP1 protein (p.E518Dfs*2) with a truncation of the last 12 amino acids (Fig. 2b). This frameshift variant has never been reported in either our cohort of 56 individuals already analyzed by whole exome sequencing or in any of the following genetic variations databases: NCBI dbSNP build138^12^, 1000 Genomes Project^13^ and NHLBI Exome Sequencing Project (ESP) Exome Variant Server^14^.

The *PSIP1* gene contains 15 coding exons (1593 nucleotides) and encodes two human transcriptional coactivators (p52 and p75) that are derived from alternatively spliced mRNAs which share a region of 325 amino acids, but show distinct coactivator properties^8^. p75 is encoded by exons 1–15 and contains 530 amino acids, while the alternatively spliced p52 is encoded by exons 1–9 and contains 333 amino acids. The c.1554_1555del, p.E518Dfs*2 mutation affects only the longer isoform (Fig. 2b).

### Gene expression analysis

**Figure 3:**
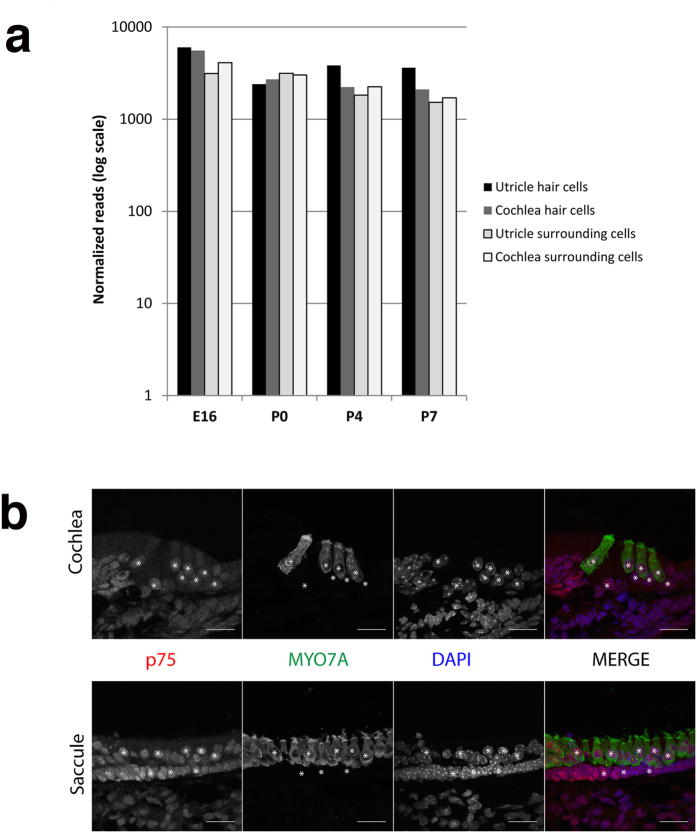
PSIP1/p75 expression in mouse inner ear. (**a**) RNA-seq expression profile of Psip1 during mouse inner ear development at embryonic age E16 and postnatal ages P0, P4 and P7. *Psip1* is expressed in hair cells and surrounding cells in both cochlea and utricle at all stages. The normalized numbers of reads are plotted on a log10 scale. (**b**) Immunolabelling of PSIP1-p75 protein (red) in the cochlea (top) and vestibule (saccule; bottom) at P6. PSIP1-p75 is expressed in the nuclei (DAPI - blue) of hair cells (co-stained with MYO7A in green) and surrounding cells in both tissues. Some nuclei are labeled * to indicate their positions.

RNA-seq on FAC-sorted hair cells and surrounding cells of the mouse cochlea and utricle showed a high and constant expression of the *Psip1* gene throughout development (from embryonic day 16 (E16) to postnatal day 7 (P7)) (Fig. 3a)^15^,^16^,^17^. Individual RNA-seq read sequences also showed that both p52 and p75 are expressed in the mouse cochlea and utricle (data not shown).

We then performed immunostaining and found PSIP1 protein located in the nuclei of all hair cells and supporting cells, of both cochlea and vestibular system (Fig. 3b). The specificity of the antibody used was verified by staining mouse embryonic fibroblasts from *Psip1*^flox/flox^ and *Psip1*^del/del^ mice (Fig. S1a,b,c).

### The frameshift deletion does not alter mRNA stability

**Figure 4:**
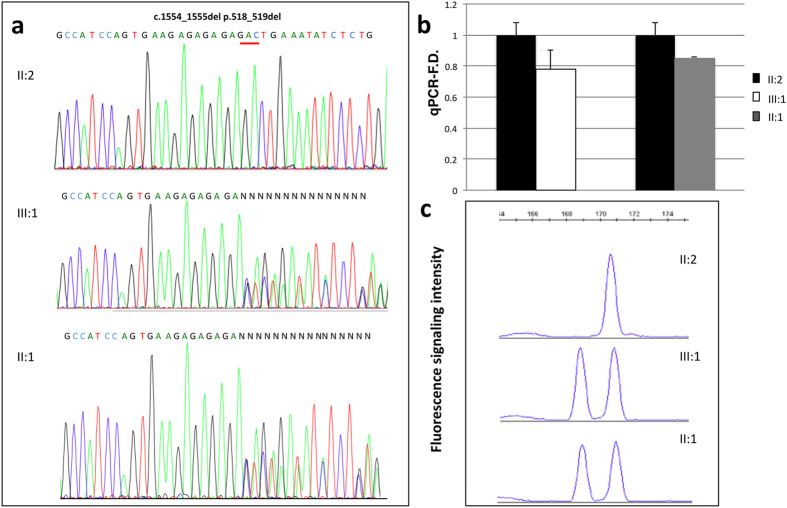
mRNA analysis of PSIP1 gene. (**a**) Chromatograms of *PSIP1* mRNA show a wild type sequence in the II:2 individual and the presence of the mutated allele in the III:1 and II:1 patients. (**b**) Quantification of *PSIP1* mRNA by real-time PCR in peripheral blood cells. Relative expression ratios represent the amount of mRNA in the *PSIP1* +/− patients (II:1;III:1) divided by that in the wild type individual (II:2). No differences were present. Assays were performed in triplicate; standard deviations are shown. (**c**) Electrophoresis followed by measurement of fluorescence intensity shows no differences in the amount of mutated and wild-type allele in patients and controls. Patients II:1 and III:1 present two peaks compared to the control (II:2) although the area under the peaks is the same.

Since the premature termination codon (PTC) is localized in the last exon of the gene, the detected deletion should not result in nonsense-mediated mRNA decay (NMD), an mRNA surveillance system that typically degrades transcripts containing PTCs^18^,^19^. Nevertheless, to understand the molecular pathogenesis of the identified frameshift deletion, we evaluated expression of normal and mutant allele transcripts. RNA from lymphoblast cell lines derived from peripheral blood was extracted from individuals III:1, II:1, and II:2. We performed Sanger sequencing of mRNA, quantitative Real-Time PCR and PCR with FAM labeled primers followed by measurement of fluorescence intensity. All these results revealed that this frameshift deletion does not lead to mRNA degradation by nonsense-mediated mRNA decay (see Fig. 4a,b) and does not produce differences in the relative amount of mutated and wild type mRNA alleles (Fig. 4c).

## Discussion

Family-based genetic mapping has facilitated the discovery of many disease-causing genes. To date, 89 NSHHL genes have been identified, divided as follows: 30 DFNA, 55 DFNB (including 9 genes overlapping with DFNA) and 4 X-linked forms. Despite this progress, the genetic basis of a large proportion of patients with DFNA remains to be elucidated. Next-generation sequencing technologies employing high-throughput exomic screening have proven to be powerful tools for discovering new disease-related genes for HHL and associated diseases^20^,^21^. The current study further demonstrates the robustness and usefulness of this approach, allowing us to identify *PSIP1* as a novel HHL candidate gene in a family affected by mild-to-moderate sensorineural hearing loss characterized by a downward slope toward the high frequencies. The identified mutation leads, once mutated, to a frameshift deletion never reported so far in population based public databases.

Very recently, this frameshift mutation has been reported in the ExAC database^22^ with a very low frequency (0.0001325). This database is largely enriched with data from many different disease consortia and only individuals affected by severe pediatric disease were removed from it. For example, a large number of genetic data on type 2 diabetes (T2D) have been included in it. The hearing phenotype has been already reported to be associated to T2D^23^. In this light, laying a T2D locus close to *PSIP1* gene, it is possible that simply due to linkage there could be an enrichment of cases with *PSIP1* mutations in the ExAC database. Thus, the use of ExAC data to calculate an allele frequency as well as to draw any molecular epidemiology conclusion could be misleading and might overestimate the role of *PSIP1* in HHL.

It is well known that PTC-bearing mRNAs are unstable when the PTC is located more than 50–55 nucleotides upstream of the last exon–exon boundary because they trigger nonproductive stalling of ribosomes^18^,^24^. Accordingly, the deletion located near the end of the transcript does not alter the *PSIP1* mRNA stability. The *PSIP1* gene encodes a transcriptional coactivator protein that has not been associated so far with any human disease. Here, for the first time, we describe a link of *PSIP1* to a human disease characterized by a very specific and peculiar hearing loss phenotype.

In mouse, PSIP1 protein is ubiquitously expressed in the ear. Accordingly, ABR findings are consistent with cochlear damage. The visual phenotype was also associated with the mutation but was extremely variable and the correlation less clear.

*PSIP1* was previously described as a player in gene regulation in the epithelial cells of the lens and in cell fate determination; it was later reported as involved in neuroepithelial differentiation and neurogenesis^9^,^25^. More recently, Elkan-Miller *et al.* showed expression in the inner ear sensory epithelia and specifically in vestibular hair cells where it is regulated by miR-135b^8^. One of the first in-depth proteomic analyses of mouse sensory cochlear epithelium by mass spectrometry confirmed the presence of *PSIP1*-P75 among cochlear proteins^26^. Taking into consideration all these findings, the identification of *PSIP1* as a HHL causative gene raises an intriguing question as to how mutations in a transcriptional co-activator expressed in many different cells types of the inner ear, in the retina and throughout the central nervous system can lead to a hearing-loss specific phenotype and variable visual phenotype not directly linked to retinal cells. The exact role of *PSIP1* in inner ear cells as well as in the visual system remains to be elucidated and further studies are needed. In this light, a mouse model recapitulating the human *PSIP1* mutation would be very useful for elucidation of a molecular mechanism as well as therapeutic intervention. Interestingly, most *Psip1/Ledfg*^−/−^ mice die perinatally while heterozygous animals showed no obvious phenotype^27^. Moreover, *PSIP1/LEDGF* has been described as a possible protector against photoreceptor degeneration, further suggesting its possible protection against degeneration of the hair cells^11^. In fact, the hearing phenotype of the *Psip1/Ledfg*^−/−^ mice has not been described; thus it is possible these animals have an as-yet-unrecognized form of late-onset form of HHL.

Finally, only a few DFNA genes show an audiogram characterised by a downward slope towards the high frequencies (i.e. DFNA9, DFNA24, DFNA22). The discovery of a new candidate gene associated with this peculiar form further increases our knowledge of the correlation between gene function and audiograms. This finding might help in predicting the phenotype on the basis of a given genotype or, on the contrary, in defining a given molecular diagnostic target on the basis of the audiogram itself.

In conclusion, we present here a disease allele in *PSIP1* gene in human thus providing new insights into the pathogenetic mechanisms underlying HHL. Although this gene was only detected in one family (common in genetic heterogeneous disorders), mutational screening of this gene in a larger collection of cases may lead to the identification of other patients/families affected by HHL, in particular those showing a downward slope towards the high frequencies at the audiograms.

## Methods

All the experiments described below were performed in accordance with relevant guidelines and regulations. Moreover, this study was approved by the Institutional Review Board (Comitato Tecnico Scientifico) of the IRCCS-Burlo Garofolo Children Hospital (Trieste, Italy) and informed consent was obtained from each participant. The protocol conformed to the tenets of the Declaration of Helsinki.

### Subject recruitment and clinical examination

A complete audiological examination of each family subject (both affected and normal hearing individuals) was carried out to clinically diagnose the hearing loss and its severity. Moreover, an ophthalmic examination was also included for three affected patients.

The study recruited an Italian family characterized by an autosomal dominant inheritance of HL. The family consists of 6 family members (the proband (III:1), his normal hearing mother (II:2) and his hearing impaired father (II:1) plus two affected (II:3,II:4) and one healthy sibling of the proband’s father (II:5). Affected subjects, classified following GENDEAF recommendations^28^, showed a late-onset, bilateral moderate sensorineural, progressive hearing loss restricted to the medium and high frequencies.

The proband (III:1), a 19-year-old male, presented with moderate, progressive, sensorineural hearing loss, restricted to the high frequencies with onset in the last year. Individuals (II:1), (II:3), (II:4) presented with adult onset, moderate but progressive sensorineural hearing loss involving medium and high frequencies. The progression of the disease led to a gradual involvement of medium frequencies and, in patients (II:1) and (II:4), to the need for hearing aids. The evaluation included a complete medical history and detailed clinical and dysmorphological examination of the patients to exclude non-genetic causes of hearing impairment (for example syphilis, toxoplasmosis, cytomegalovirus, injuries, etc.) or the presence of SHHL, but no other relevant feature was detected. Pure tone audiometry and otoscopy were performed for all six individuals by standard procedures. As regards the ophthalmic phenotype, VEPs analysis was performed in the proband (III:1) and two other affected individuals (II:1, II:3) showing slightly differences. Examinations were carried out in November 2014.

### Targeted and whole-exome sequencing

The proband (III:1), his parents (II:1,II:2) and other two relatives (II:3, II:5) were recruited for sequencing analysis. A total of 96 HHL genes were analyzed by targeted sequencing as previously described^7^. The whole exome sequencing was performed at the Europe Life Technologies Ion AmpliSeq™ Exome Certified Service Providers, CRIBI Sequencing Core, University of Padua, Italy^29^. The DNA libraries were prepared employing the Ion AmpliSeq™ Exome Kit and were then sequenced by the Ion Proton™ System (Life Technologies, CA, USA), according to standardized procedures. Reads mapping and variant calling were performed by the Ion TorrentSuite™v4.0 software (Life Technologies, CA, USA) set up with standardized parameters. Single Nucleotide Variations (SNVs) and small Insertions and Deletions (INDELs) were provided into a Variant Call Format (VCF) version 4.1^30^. SNVs and INDELS were annotated using the most updated version of ANNOVAR^31^. The SNVs/INDELs were filtered out using the following exclusion criteria: 1) SNVs leading to synonymous amino acids substitutions not predicted pathogenic by any disease predictor tools (SIFT, Polyphen2, MutationTaster) and not affected highly conserved residues (PhyloP); 2) SNVs/INDELs called in off-target regions. Further, a comparison between identified genetic variants and data reported in NCBI dbSNP build138^12^ as well as in 1000 Genomes Project^13^ and NHLBI Exome Sequencing Project (ESP) Exome Variant Server^14^ was carried out. In this light, taking into account that a lower MAF cutoff of 0.1% is helpful for dominant disorders, as the estimated prevalence of the disorder (generally well below 0.1%) provides an upper bound on the MAF, variants above this cutoff were excluded from the analysis. In order to specifically identify the putative pathogenic mutation, we performed a filter designed to select those variants segregating in the family in accordance to the hypothesized disease dominant pattern of transmission. Therefore the SNVs/INDELs were filtered using VCFtool^30^, in agreement with a dominant inheritance model, namely selecting all variants called in heterozygous state in affected individuals and simultaneously not called in healthy subjects. We manually investigated the raw sequence reads for all the candidate pathogenic variants using the Integrative Genomics Viewer (IGV) with the purpose of excluding likely false positive calls due to read misalignment^32^. Finally, the most likely identified disease-causing SNVs/INDELs were analyzed by direct Sanger sequencing on a 3500 Dx Genetic Analyzer (Life Technologies, CA, USA), using ABI PRISM 3.1 Big Dye terminator chemistry (Life Technologies, CA, USA) according to the manufacturer’s instructions. Sanger sequencing was employed both to exclude false positive SNVs calling and to perform the segregation analysis within the family.

### Mutation pathogenicity assessment

The impact of missense mutations on the protein structure was assessed using three *in silico* “pathogenicity” predictor tools, namely, Polyphen-2, MutationTaster, and SIFT 33,34,35. Moreover, evolutionary conservation of nucleotides across species was evaluated by the PhyloP algorithm^36^. PhyloP score is a measurement of the conservation of each DNA base referrenced to information from 44 vertebrates; this score has been employed to categorize the SNVs involving conserved nucleotides, referring to LJB_PhyloP scores reported in the dbNSFP database^37^. The SNVs were categorized according the following criteria: LJB_PhyloP > 0.95 nucleotide = conserved, LJB_PhyloP < 0.95 nucleotide = not conserved. In our view, the substitution of a phylogenetically conserved nucleotide is a predictor of deleteriousness, namely a variation that reduces the organism’s fitness, and is strongly related with molecular pathogenicity.

Both The Human Gene Mutation Database (HGMD)^38^ and OMIM^39^ were also consulted to assess whether identified variants were novel or already associated with a phenotype.

### Immunohistochemistry

Inner ears of P6 and P0 CD1 mice were collected, fixed in 4% formaldehyde and cryosectioned (7- to 10-μm-thick sections). MEF cells were obtained from A.N. Engelman at the Dana-Farber Cancer Institute^40^. A microwave antigen retrieval technique was applied (H-3300, Vector Laboratories) prior to permeabilization/blocking in 1X PBS + 0.05% triton + 8% normal goat serum for 1 h at 22 °C. The sections were then incubated with primary antibodies overnight at 4 °C and secondary antibodies for 1 h in blocking solution at 22 °C. Stained slices and tissues were mounted with ProLong Gold Antifade Reagent with DAPI (Invitrogen).

Antibodies used were mouse monoclonal anti-MYO7A (1/2000) (Developmental Studies Hybridoma Bank, University of Iowa #138–1) and rabbit anti-LEDGF/PSIP1 (1/25) (Cell Signaling C57G11). Tissues and cells were then viewed with a confocal microscope (Olympus Fluoview 1000). Images shown are maximum value projections of brightfield and fluorescence stacks.

### RT-PCR

RNA extraction from peripheral blood was performed using a semiautomatic extractor (QIAsymphony, Qiagen, Germany) and quantified on a Nanodrop ND-1000 spectrophotometer (NanoDrop Technologies, Wilmington, DE, USA). 0.5 μg of total RNA was used for cDNA synthesis using the ThermoScript Reverse Transcriptase kit (Invitrogen). PCR was performed on cDNA using the primers (Psip1-1F_5′/3′: AACAAACAGGGTCAAAGACT; Psip1-1R: AGTGTTCTCTATATTCCAGG) and analyzed by direct sequencing using an ABI PRISM BigDye Terminator Cycle 3.1 Sequencing Ready Reaction Kit and a 3500 Dx Genetic Analyzer (Applied Biosystems, Foster City, CA).

### Real-time qRT-PCR

Quantitative real time PCR (qPCR) for the *PSIP1* gene was performed using standard PCR conditions in a 7900HT Fast Real Time PCR (Life Technologies) with Power SYBR Green PCR Master Mix (Life Technologies). Gene-specific primers were designed by using PRIMEREXPRESS software (Applied Biosystems). The thermal cycling conditions were composed of 50 °C for 2 min followed by an initial denaturation step at 95 °C for 10 min, 40 cycles at 95 °C for 15 s, 59 °C for 1min and 72 °C for 30 s. The experiments were performed in biological triplicate. Expression levels were standardized to β-actin gene expression and all the data were analyzed using the 2^–ΔΔCT^ Livak Method^41^.

### Analysis RNA yield

cDNA was amplified by PCR with a pair of primers (Psip1-1F_5′/3′: AACAAACAGGGTCAAAGACT; Psip1-1R: AGTGTTCTCTATATTCCAGG) and the Psip1-1F was labeled with fluorescein (5′6-FAM). The amount and the size of the PCR products were determined by electrophoresis followed by measurement of fluorescence intensity with an automated genetic analyzer (ABI PRISM3130).

## Additional Information

**How to cite this article**: Girotto, G. *et al.* *PSIP1/LEDGF*: a new gene likely involved in sensorineural progressive hearing loss. *Sci. Rep.* **5**, 18568; doi: 10.1038/srep18568 (2015).

## Supplementary Material

Supplementary Information
