# Supplementary material for: PSIP1/LEDGF: a new gene likely involved in sensorineural progressive hearing loss
Source: Sci Rep. 2015 Dec 22;5:18568. doi: 10.1038/srep18568 (PMC4686969; doi:10.1038/srep18568)
Supplement: Supplementary Information [file srep18568-s1.pdf]

PSIP1/LEDGF: a new gene likely involved in sensorineural progressive hearing loss

Giorgia Girotto, Déborah I. Scheffer, Anna Morgan, Diego Vozzi, Elisa Rubinato, Mariateresa Di Stazio, Enrico Muzzi, Stefano Pensiero, Anne B. Giersch, David P. Corey, Paolo Gasparini

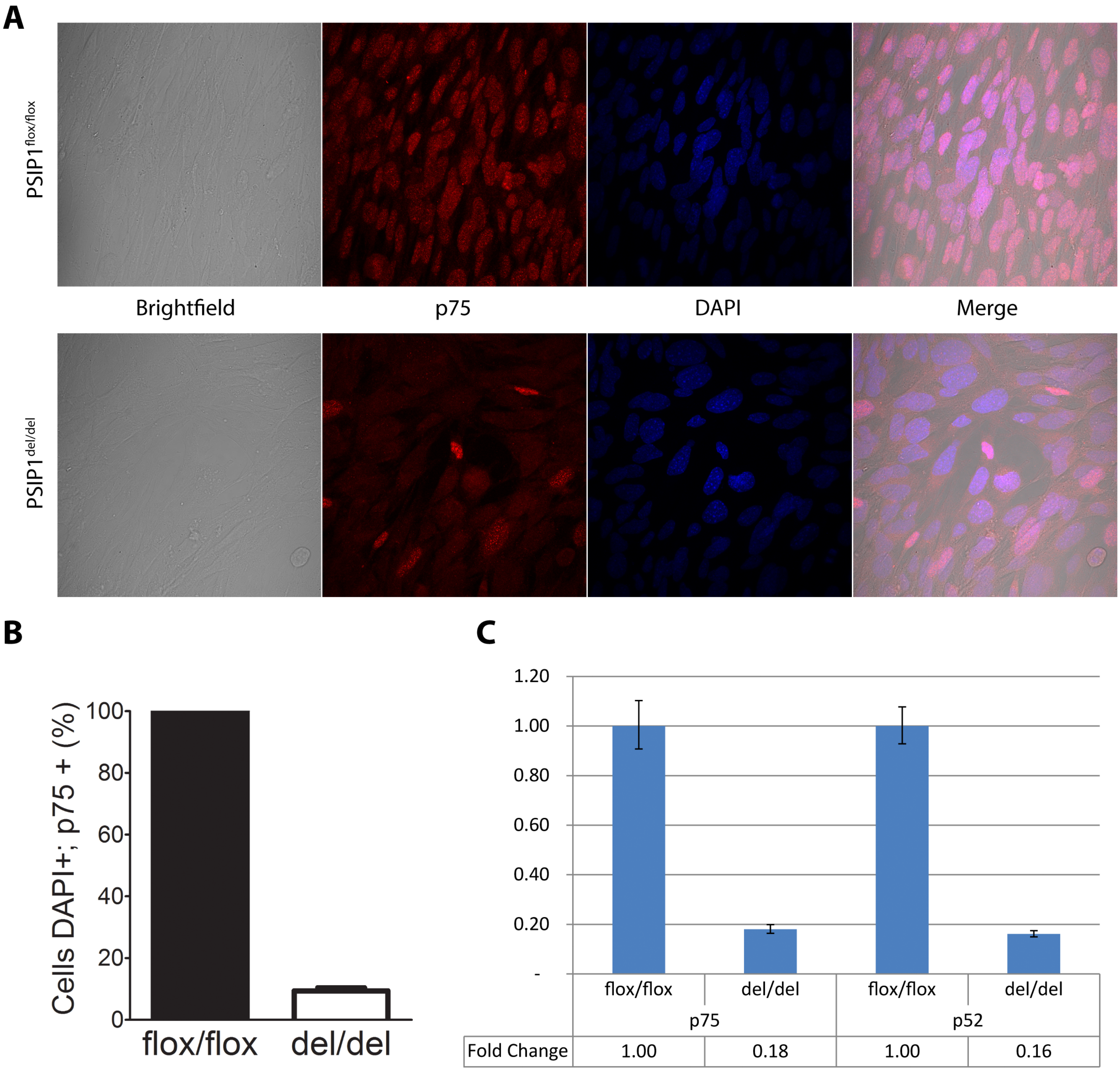

Figure S1 : Validation of p75 antibody specificity.

A. p75 immunostaining in mouse embryonic fibroblasts derived from Psip1$^{flox/flox}$ animals. All control cells (Psip1$^{flox/flox}$) express p75 when only a few CRE recombined cells (Psip1$^{del/del}$) retain the staining. Cells were obtained from Dr Engelman lab (Shun et al, 2007).

B. Quantification of the number of mouse embryonic fibroblasts expressing p75. Cells were counted using DAPI and the percentage of p75 positive nuclei was represented. 100±0% of Psip1$^{flox/flox}$ cells expressed p75 when 9.4±1% of Psip1$^{del/del}$ did.

C. Quantitative PCR of p52 and p75 isoforms in Psip1$^{flox/flox}$ (flox/flox) and Psip1$^{del/del}$ (del/del) cells. The expression level relative to control cells is plotted (fold change). Standard errors from duplicate experiments are shown. The 18% residual expression of p75 accounts for the residual p75 labelled cells in A, B.

PSIP1/LEDGF: a new gene likely involved in sensorineural progressive hearing loss

Giorgia Girotto, Déborah I. Scheffer, Anna Morgan, Diego Vozzi, Elisa Rubinato, Mariateresa Di Stazio, Enrico Muzzi, Stefano Pensiero, Anne B. Giersch, David P. Corey, Paolo Gasparini

	III:1	II:1	II:3	II:5
**Total aligned base reads**	7.991.454.803	5.750.542.769	6.444.727.040	6.336.834.597
**Total base reads on target**	7.579.951.951	5.449.964.467	6.090.441.616	5.989.209.463
**Bases in target regions**	57.742.646	57.742.646	57.742.646	57.742.646
**Percent base reads on target**	94,85%	94,77%	94,50%	94,51%
**Average base coverage depth**	131,3	94,38	105,5	103,7
**Target base coverage at 1x**	98,43%	98,19%	98,07%	98,31%
**Target base coverage at 20x**	91,28%	87,74%	88,21%	89,12%
**Target base coverage at 100x**	52,71%	36,93%	42,52%	41,74%
**Target base coverage at 500x**	0,99%	0,16%	0,31%	0,24%
**Target bases with no strand bias**	77,23%	75,04%	67,70%	72,57%

Supplementary Table 1
Coverage Analysis Report of the Whole Exome Sequencing analysis: a detailed summary of the exome sequencing bases coverage. It takes into account the overall reads either general and on target: Total aligned base reads and Total base reads on target. The overall amount of bases mapped on the target region: Bases in target regions and Average base coverage depth. The minimum portion of target regions covered at least at 1X, 20X,100X and 500X: Target base coverage at 1x, Target base coverage at 20x, Target base coverage at 100x, Target base coverage at 500x. Finally the strand bias (genotype discordance between the positive strand and negative strand) effect has been evaluated: Target bases with no strand bias.
